# In vivo contrast‐enhanced microCT for the monitoring of mouse thoracic, lumbar, and coccygeal intervertebral discs

**DOI:** 10.1002/jsp2.1058

**Published:** 2019-06-26

**Authors:** Remy E. Walk, Simon Y. Tang

**Affiliations:** ^1^ Department of Biomedical Engineering Washington University in St. Louis St. Louis Missouri; ^2^ Department of Mechanical Engineering and Materials Science Washington University in St. Louis St. Louis Missouri; ^3^ Department of Orthopaedic Surgery Washington University in St. Louis St. Louis Missouri

**Keywords:** aging, Contrast‐enhanced microCT, intervertebral disc, mouse model

## Abstract

Mouse models are often used for studies of intervertebral disc (IVD) homeostasis and degeneration, yet the relatively small size of the IVD poses challenges for noninvasive, longitudinal imaging modalities. The recently developed contrast‐enhanced microCT (CEμCT) using Ioversol has been successful in detecting degenerative changes in the murine IVD ex vivo at the micrometer scale. Further leveraging the superior biocompatibility of Ioversol as a contrast agent, we demonstrate the in vivo use of this CEμCT technique to examine IVDs at multiple spinal sites. Ioversol was administered via tail vein injection (TVI) in growing and adult male FVB/NJ mice (n = 5 /group). The animals were anesthetized and underwent in vivo micro‐computed tomographic (microCT) at the coccygeal (CC5/CC6), lumbar (L5/6), and thoracic (T12/T13) IVDs. TVI of Ioversol was well‐tolerated by all animals. As Ioversol filtered through the kidneys and accumulated in the bladder, the attenuations of the mouse bladder and kidneys increased due to the high molecular weight of Ioversol, confirming that the Ioversol is biological available. Average IVD attenuations increased 3%‐15% following TVI (ANOVA; *P* < .01). The presence of Ioversol in the IVD combined with high‐resolution microCT allow for nondestructive visualization of structural features of the IVD. These results demonstrate CEμCT with Ioversol as a viable strategy for the in vivo monitoring of multiple mouse IVDs during degeneration, disease, and injury.

## 1 INTRODUCTION

Low back pain is one of the most common causes of disability.^1^ With intervertebral disc (IVD) degeneration being a leading contributor to the development of low back pain, there is a need for a better understanding of the biological and structural changes of the IVD that occur with the disease and injury.^2^ Mouse models are useful for studying the molecular mechanisms of IVD biology because of the number of genetically modified models and established techniques to induce degeneration.^3^ However, resolving the micrometer‐scale structural features of the mouse IVD remains a significant challenge even with state‐of‐the‐art small animal microMRI technologies.^4^

Micro‐computed tomographic (microCT) techniques have outstanding spatial resolution for the volumetric imaging of mineralized tissues in rodents.^5^ However, since nonmineralized tissues do not attenuate X‐Rays, a contrast agent is required to differentiate the soft tissue of interest. Although ionic and heavy‐metal and ionic contrast‐agents have shown to improve the X‐ray attenuation of IVDs for ex vivo imaging^6^, ^7^, the nonionic, hydrophilic, and highly biocompatible nature of Ioversol make it particularly suited for in vivo use.^8^ Ioversol has been used clinically as a diagnostic radiographic contrast agent. With a high molecular weight, 807.115 g/mol, Ioversol reduces the x‐rays that can pass through the tissue and increases the attenuation of blood vessels, organs, and other nonmineralized tissues. We have previously reported on a contrast‐enhanced microCT (CEμCT) technique using Ioversol to monitor and quantify IVD degeneration in vitro and ex vivo.^9^, ^10^ Extending this work further, the objective of this study is to explore and validate the in vivo use of CEμCT technique using Ioversol to examine intervertebral discs at multiple spinal sites using growing and adult FVB/NJ mice.

## 2 METHODS

### 2.1 Animals

All procedures were performed with the approval of Washington University School of Medicine Institutional Animal Care and Use Committee. Male FVBN/J mice at two age groups, 3‐ (growing) and 8‐ (adult) month old, were used in this study (n = 5 / group). Wild‐type mice were selected for this study to examine the diffusion of Ioversol into the IVD under healthy conditions. Mice were kept in standard husbandry conditions with 12‐hour light/dark cycles as described by Jackson Labs (https://www.jax.org/jax-mice-and-services/customer-support/technical-support/breeding-and-husbandry-support/mouse-room-conditions). Mice were injected with 8 mL/kg of 350 mg/mL of Ioversol (OptiRay 350; Guerbet, St. Louis, Missouri) which reflects veterinary recommendations for a mouse at the highest available concentration of Ioversol. Mice were placed in a restrainer on a heat pad and lidocaine was applied to the tail. A 29G syringe was used to inject Ioversol into the lateral tail vein of the mouse. Mice were then anesthetized by isoflurane prior to microCT imaging.

### 2.2 MicroCT imaging

MicroCT imaging was conducted on a VivaCT40 system (Scanco Medical, CH) at 10 μm voxel size (45 kVp, 177 uA, 116 ms integration time). Our preliminary studies showed that at least 20 minutes is required for TVI‐administration of Ioversol to diffuse into the intradiscal space to increase the attenuation of the tissue. We observed that the bioavailability of Ioversol diminish significantly 2‐hours post‐TVI.

**Figure 1:**
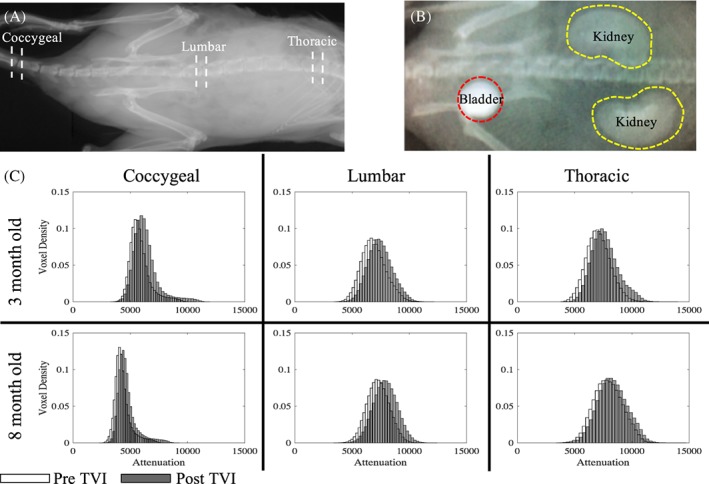
A, A radiograph of mouse indicating the CC5/CC6, L5/L6, and T12/T13 IVDs that were scanned for this study. B, Bioavailability of Ioversol is confirmed by the increased attenuation of the bladder and kidneys approximately 15 minutes post‐TVI. C, Typical histograms of pre‐TVI and post‐TVI scans of the three spinal levels in 3‐ and 8‐month old mice. Injection with Ioversol shifts the histogram to the right and reduces the number of low attenuating voxels

To determine the optimum time to achieve the maximum attenuation, each site is scanned at several timepoints following the injection. During the 110 minutes scanning period, three intervertebral levels (coccygeal [CC], lumbar [L], and thoracic [T]; Figure 1A) were scanned at three timepoints to observe the diffusion of Ioversol through different levels of the spinal column. The CC5/CC6, L5/L6, and T12/T13 IVDs were imaged for this study. CC5/CC6 was imaged at 20, 50, and 80 minutes; L5/L6 at 30, 60, and 90 minutes; and T12/T13 at 40, 70, and 100 minutes after injection. A scan at each site took approximately 8 minutes. The staggered timepoints at different sites were necessary to account for the times required for source and detector repositioning. A scan of each level was taken prior to injection with Ioversol to determine a baseline attenuation. The maximum attenuations from the three time points are determined and tabulated.

### 2.3 Image processing

The raw data was exported to DICOM for further analysis using a custom MATLAB program (https://github.com/WashUMRC/ContouringGUI). Following an initial median filter (weight = 0.8, radius = 7), vertebral bodies were used as landmarks to remove the surrounding soft tissue. Contours were drawn around the outer edge of the vertebral bodies and morphed using linear interpolation. The bone was excluded to generate a mask of the IVD. The threshold was verified by visual inspection to ensure that all voxels containing bone were removed from the mask. The mask undergoes a morphological open followed by a morphological close to smooth edges and fill voids. From the mask of the whole disc, volume and average attenuation were calculated. The volume was determined from the total number of voxels included in the mask. The mean attenuation was taken as the average 16‐bit grayscale value of the voxels contained within the mask. The mean attenuation of scans following TVI of Ioversol was normalized to their respective baseline scans. The ratio of the mean attenuation of the post‐TVI scan (DI) to the pre‐TVI (DI_0_) was calculated for both groups at all levels. Disc heights and visualizations were obtained using OsiriX (Pixmeo, Geneva, Switzerland). Disc height represents the average disc height across five evenly spaced points along the mid‐sagittal plane of the IVD.

### 2.4 Statistics

A 3‐way ANOVA was performed to determine the effect of age, spinal level, and Ioversol‐injection on the attenuations of the IVDs. Statistical were conducted using RStudio 1.1.453 (RStudio, Boston, Massachusetts).

## 3 RESULTS

Tail vein injection (TVI) of Ioversol was well‐tolerated by the mice with no adverse events observed up to 4 weeks post injection. Shortly following TVI, the bladder and kidney became attenuating features in the microCT‐scout view, confirming the metabolism and bioavailability of Ioversol (Figure 1B).

**Figure 2:**
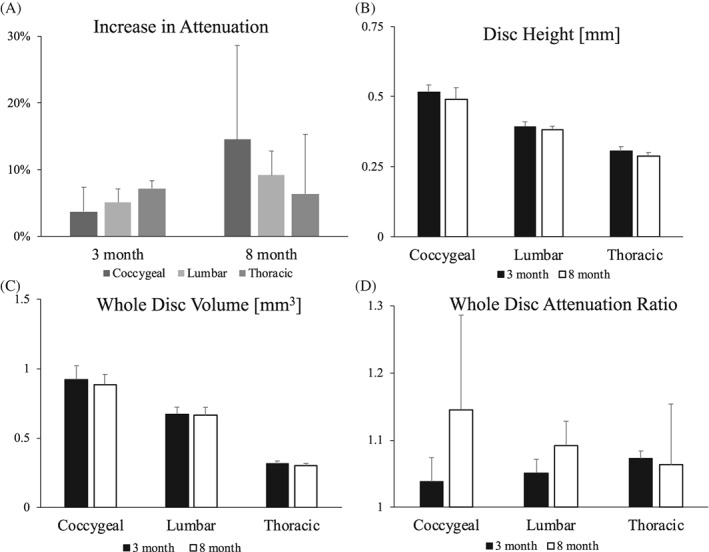
A, TVI significantly increased the attenuations of the IVDs in the 3‐ and 8‐month animals (3‐way ANOVA; *P* < .01). B and C, The 3D data enables the unbiased quantification of disc height and whole disc volume. D, The whole disc attenuation ratio (DI/DI_0_) increased in the adult lumbar IVDs

The post‐TVI scans showed increased attenuations of the IVD regions compared with the pre‐TVI scans. Qualitative inspection also showed that the IVD attenuation increased compared to other soft tissues in the IVD's vicinity. Quantitative comparisons of the voxel densities versus attenuations showed that the entire intradiscal region shifts towards elevated values (Figure 1C), with the average increase ranging from 3% to 15% (*P* < .01; Figure 2A,D). Moreover, there is a reduction in the number of low attenuating voxels. Taken together, the Ioversol delivered by TVI was biologically available, and the Ioversol was transported to the IVD where it was preferentially taken up compared to the surrounding soft tissues.

**Figure 3:**
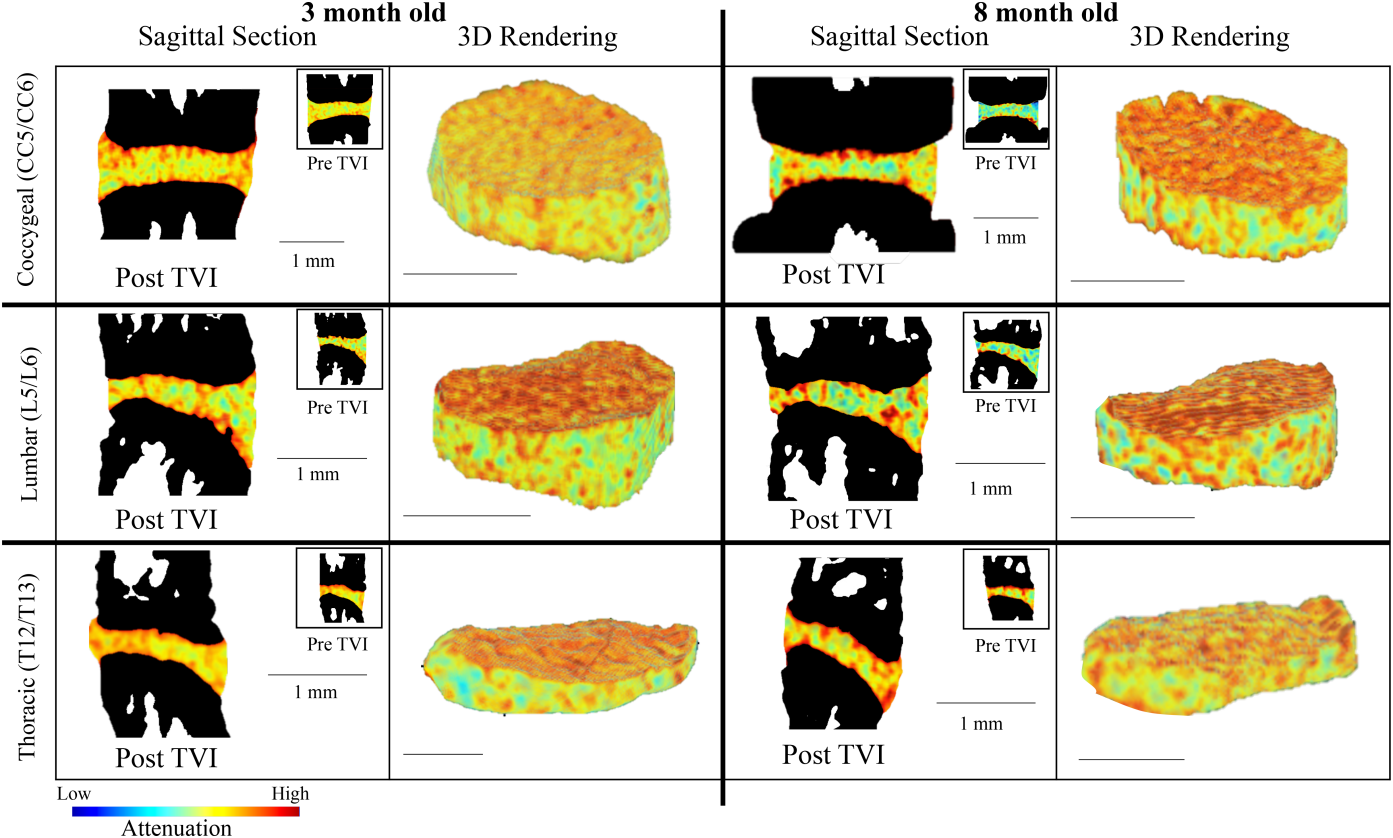
2D and 3D renderings of pre‐ and post‐TVI of the CC5/CC6, L5/L6, and T12/T13 IVDs in 3‐ and 8‐month old mice. The increased attenuations after TVI allowed for contouring of the IVD tissue boundary for the accurate 3D reconstructions of the IVD

In order to explore the time‐dependence of Ioversol retention in the IVD, we monitored each intervertebral disc at three timepoints as technically allowed by the microCT system. Mean attenuation varied by less than 5% between the three post‐TVI scans (data not shown). Increased attenuation following injection with Ioversol was observed across all levels and ages (Figure 2A, D). At the coccygeal level, in both 3‐ and 8‐month old mice, maximum attenuation was achieved 50 minutes after injection with Ioversol and maintained throughout the scanning period of the tail disc (50‐90 minutes). The L5/L6 disc reached maximum attenuation at 30 minutes post‐TVI in 3‐month old mice and in 8‐month old mice. At the thoracic level, a maximum attenuation achieved 40 minutes after TVI in both 3‐ and 8‐month old mice that is maintained throughout the scanning of the T12/T13 IVD (40‐110 minutes). Morphological parameters such as disc height and volume can be directly quantified from the microCT images (Figure 2B, C). No significant differences between ages were observed. Following contouring and segmentation, three‐dimensional visualizations of the IVDs were rendered (Figure 3).

## 4 DISCUSSION

To date, this is the first study to describe the in vivo use of CEμCT for the imaging of the mouse intervertebral disc. This approach was previously demonstrated in several ex vivo rodent models of IVD degeneration, and it achieved superior spatial resolution compared with microMRI.^9^, ^10^ By delivering Ioversol through tail‐vein injection at a physiologically compatible dose, Ioversol was biologically available and accumulated preferentially in the IVD to increase the attenuation of all the scanned IVDs. We obtained disc height and volume measures of the IVDs expected from the aging mouse IVD.^6^, ^11^, ^12^

The TVIs were well‐tolerated by all mice with no adverse consequences, suggesting that the technique would be suitable for longitudinal monitoring. However, it is worth noting that Ioversol is metabolized through the mouse quickly, and we observe declines in IVD and kidney signal as early as 120 minutes post‐TVI. As the natural course of urinary homeostasis, the bladder attenuation increases over time until the Ioversol is expelled. This accumulation of contrast‐agent in the bladder could occasionally introduce beam‐hardening artifacts if the IVD of interest is localized near the bladder, and we would suggest users of this approach to be cognizant of the anatomical variations and metabolic rate of the mouse model. We observed that time required to achieve maximum attenuation depended on age of the animal and spinal level, and it may be important to optimize injection and imaging parameters for animals of different sex, strain, and backgrounds. Here, we were limited by the scan time and time for detector repositioning for optimizing post‐TVI scan time; future work focused on a single level would allow additional timepoints to be investigated for further refinement of the optimal time between injection and post‐TVI scan. The attenuation signals are influenced by motion and partial volume effects, and this is particularly evident in the thoracic IVD due to the narrow intradiscal space (approximately 100‐200 μm disc height) and proximity to the lungs during breathing. Motion artifacts and partial volume effects may have contributed to the variation in the attenuation response post‐TVI. As a result, thoracic IVDs may not be suited for this technique. Yet the lumbar and coccygeal vertebrae did not exhibit motion artifacts, suggesting that motion was not a factor at these sites. It is worth noting that alterations in endplate porosity and hydration levels may also influence the infiltration of Ioversol and attenuation of the IVD following TVI. Our approach here accounts for these variations by scanning each spinal site at multiple timepoints to determine the time between injection and imaging to maximize the diffusion of Ioversol into the IVD. If only one or two adjacent IVDs were monitored, the signal‐to‐noise can be improved through increasing integration and/or exposure times. Variations due to TVI contrast‐agent delivery may be mitigated with blood vessel cannulation in the future.

The current CEμCT technique allows for the high‐resolution imaging of the mouse IVD in vivo at spatial resolutions at tens of microns,^4^ and thus can quantify the structural characteristics of the IVD with greater precision and fidelity. In addition, this approach allows for the imaging of multiple spinal sites with a single set up. TVI of Ioversol was well‐tolerated by the mice and demonstrates the feasibility this technique for longitudinal studies. Previous studies have utilized in vivo microCT for longitudinal studies with no adverse events reported due to radiation.^13^, ^14^ CEμCT will be a valuable strategy for in vivo monitoring of IVD injury and degeneration across multiple levels in mouse models.

## CONFLICT OF INTEREST

All authors state they have no conflicts of interest.

## AUTHOR CONTRIBUTIONS

Remy Walk

1. Performed experiments
2. Analysis and interpretation of the data
3. Drafted manuscript
4. Approved final version of manuscript

Simon Tang

1. Conceived and designed research
2. Analysis and interpretation results of experiments
3. Edited and revised manuscript
4. Approved final version of manuscript

## References

[1] Hoy D , Bain C , Williams G , et al. A systematic review of the global prevalence of low back pain. *Arthritis Rheum* 2012;64(6):2028‐2037. 10.1002/art.34347 22231424

[2] Luoma K , Riihimäki H , Luukkonen R , Raininko R , Vilkari‐Juntura E , Lamminen A . Low back pain in relation to lumbar disc degeneration. Spine. 2000;25(4):487‐492. 10707396 10.1097/00007632-200002150-00016

[3] Lotz J . Animal model of intervertebral disc degeneration: lessons learned. Spine. 2004;29(23):2742‐2750. 15564923 10.1097/01.brs.0000146498.04628.f9

[4] Kagadis GC , Loudos G , Katsanos K , Langer SG , Nikiforidis GC . In vivo small animal imaging: current status and future prospects. Med Phys. 2010;37(12):6421‐6442. 21302799 10.1118/1.3515456

[5] Bouxsein M , Boyd S , Christiansen B , Guldberg R , Jepsen K , Müller R . Guidelines for assessment of bone microstructure in rodents unse micro‐computed tomography. J Bone Miner Res. 2010;25(7):1468‐1486. 20533309 10.1002/jbmr.141

[6] Bhalla S , Lin KH , Tang SY . Postnatal development of the murine notochord remnants quantified by high‐resolution contrast‐enhanced microCT. Sci Rep. 2017;7(1):13361. 29042621 10.1038/s41598-017-13446-5 PMC5645339

[7] Newton M , Hartner S , Timmons S , et al. Contrast‐enhanced μCT of the intervertebral disc: a comparison of anionic and cationic contrast agents for biochemical and morphological characterization. J Orthop Res. 2017;35(5):1067‐1075. 27415967 10.1002/jor.23364

[8] McClennan BL . Ionic versus nonionic contrast media: safety, tolerance, and rationale for use. Urol Radiol. 1989;11(4):200‐202. 2692266 10.1007/BF02926515

[9] Lin K , Wu Q , Leib D , Tang S . A novel technique for the contrast‐enhanced microCT imaging of murine intervertebral discs. J Mech Behav Biomed Mater. 2016;63:66‐74. 27341292 10.1016/j.jmbbm.2016.06.003 PMC4983496

[10] Lin K , Tang S . The quantitative structural and compositional analyses of degenerating intervertebral discs using magnetic resonance imaging and contrast‐enhanced micro‐computed tomography. Ann Biomed Eng. 2017;45(11):2626‐2634. 28744842 10.1007/s10439-017-1891-8 PMC5665707

[11] Dahia C , Mahoney E , Durrani A , Wylie C . Postnatal growth, differentiation, and aging of the mouse intervertebral disc. Spine. 2009;34(5):447‐455. 19247165 10.1097/BRS.0b013e3181990c64

[12] Holguin N , Aguilar R , Harland R , Bomar B , Silva M . The aging mouse partially models the aging human spine: lumbar and coccygeal disc height, composition, mechanical properties, and Wnt signaling in young and old mice. J Appl Physiol. 2014;116(12):1551‐1560. 24790018 10.1152/japplphysiol.01322.2013 PMC4064379

[13] Laperre K , Depypere M , van Gastel N , et al. Development of micro‐CT protocols for the in vivo follow‐up of mouse bone architecture without major radiation side effects. Bone. 2011;49(4):613‐622. 21763477 10.1016/j.bone.2011.06.031

[14] Detombe S , Dunmore‐Buyze J , Petrov I , Drangova M . X‐ray dose delivered during a longitudinal micro‐CT study has no adverse effect on cardiac and pulmonary tissue in C57BL/6 mice. Acta Radiol. 2013;54(4):435‐441. 23436828 10.1177/0284185113475608

